# Perfectionism Dimensions and Longitudinal Depressive Symptom Profiles in Adolescents: Distinguishing Evaluative Self-Criticism from Personal Standards

**DOI:** 10.3390/bs16091678

**Published:** 2026-09-17

**Authors:** Meng Yuan, Yuheng Huang

**Affiliations:** College of Education, Capital Normal University, No. 105 West Third Ring Road North, Haidian District, Beijing 100048, China; 2240402049@cnu.edu.cn

**Keywords:** perfectionism, self-critical perfectionism, personal standards, depressive symptoms, adolescents, longitudinal profiles

## Abstract

Perfectionism is multidimensional, yet it remains unclear whether evaluative self-criticism and high personal standards differentially identify adolescents with unfavorable depressive symptom profiles. Using data from a baseline sample of 1150 adolescents aged 14 to 19 years and six monthly follow-up assessments, a three-class quadratic growth mixture model identified low-stable, high-decreasing, and increasing/high-escalating profiles among 1129 trajectory-eligible participants. Three-Step Approach(R3STEP) models entered self-critical perfectionism, personal standards perfectionism, age, sex, rural/urban source, and highest parental education simultaneously; a sensitivity model additionally included baseline depressive symptoms. Self-critical perfectionism was associated with both non-low-stable profiles and remained significant after baseline symptom adjustment, although estimates were attenuated. Personal standards perfectionism showed no consistent association. Direct coefficient contrasts indicated stronger self-critical than personal-standards effects for both non-low-stable profiles in the primary model, but these between-predictor differences were no longer significant after baseline Center for Epidemiologic Studies Depression Scale (CES-D) adjustment. A targeted sensitivity analysis excluding parental education as a covariate yielded the same substantive pattern for self-critical perfectionism. The findings provide a person-centered extension of multidimensional perfectionism-depression research while also indicating that the two Child and Adolescent Perfectionism Scale(DPSCA) subscales showed only modest internal consistency and imperfect absolute factorial fit in this sample.

## 1. Introduction

Depressive symptoms can become more visible, impairing, and differentiated across individuals during adolescence. They are associated with difficulties in academic functioning, peer relationships, family adjustment, and later psychological health (Thapar et al., 2012). A central question for personality and behavioral science is whether baseline vulnerability dimensions help distinguish adolescents who remain low in symptoms from those who show elevated or worsening symptom profiles over time (Batterham et al., 2026; Jamil et al., 2025).

Person-centered longitudinal research indicates that adolescent depressive symptoms do not follow a single course. Many adolescents maintain low symptom levels, whereas smaller groups show elevated, decreasing, persistent, or increasing patterns. Average-level analyses may obscure these subgroups; latent class growth and growth mixture models are, therefore, useful for identifying heterogeneous symptom courses (Batterham et al., 2026; Pakhomova et al., 2026; Reck et al., 2024). A further task is to identify baseline characteristics that differentiate membership in these profiles.

Perfectionism is a relevant vulnerability domain, but it is not unitary. Multidimensional accounts distinguish evaluative forms involving harsh self-judgment, concern over mistakes, and contingent self-worth from forms centered on high personal standards and achievement striving (Dunkley et al., 2006; Hewitt & Flett, 1991; Stoeber & Otto, 2006). Self-critical perfectionism reflects punitive self-evaluation and perceived failure, whereas personal standards perfectionism reflects demanding goals. Their psychological implications may differ, particularly when high standards are not accompanied by harsh self-criticism.

This distinction may be especially salient during adolescence, when academic evaluation, peer comparison, parental expectations, and identity-related challenges intensify (Flett et al., 2016; Thapar et al., 2012). Adolescents high in self-critical perfectionism may interpret ordinary setbacks as evidence of inadequacy, whereas personal standards may reflect achievement motivation unless coupled with concern over mistakes or fear of failure (Dunkley et al., 2006; Limburg et al., 2017; Smith et al., 2021). Examining the two dimensions simultaneously can therefore clarify whether depressive symptom profile membership is associated with evaluative self-criticism, standards-related striving, or both.

Meta-analytic evidence links perfectionism, particularly perfectionistic concerns, with depressive symptoms and broader psychopathology (Limburg et al., 2017). A youth-specific meta-analysis of participants aged 6–24 years likewise found a moderate association between perfectionistic concerns and depressive symptoms and a generally weaker pattern for perfectionistic strivings across psychopathology outcomes (Lunn et al., 2023). Longitudinal evidence suggests that perfectionism can prospectively predict depressive symptoms and that depressive symptoms may also predict later perfectionism, indicating reciprocal processes (Smith et al., 2021). Stress generation and social disconnection are among the proposed mechanisms (Smith et al., 2020). Youth research similarly connects perfectionism with distress and maladjustment and suggests that perfectionism and depressive symptoms may co-develop (Flett et al., 2016; Vaillancourt & Haltigan, 2018).

Two gaps remain. First, less is known about whether baseline perfectionism dimensions distinguish membership in empirically derived depressive symptom profiles across repeated assessments. Second, self-critical and personal standards dimensions should be entered together so that each estimate reflects its association, conditional on the other dimension. Baseline depressive symptoms must also be considered because perfectionism and current symptoms overlap conceptually and empirically (Limburg et al., 2017; Smith et al., 2021).

### Hypotheses

The study used six monthly follow-up assessments to identify depressive symptom profiles and test whether baseline perfectionism dimensions predicted profile membership. We expected higher self-critical perfectionism to be associated with both non-low-stable profiles, relative to the low-stable profile. Personal standards perfectionism was expected to show weaker or less consistent associations. Age, sex, rural/urban source, and highest parental education were examined as secondary predictors of profile membership, and a sensitivity model additionally included baseline depressive symptoms.

## 2. Materials and Methods

### 2.1. Overview of Study Design and Analytic Approach

This school-based longitudinal study used growth mixture modeling to identify heterogeneous profiles of depressive symptoms across six monthly follow-up assessments. After the profile solution was selected, Three-Step Approach(R3STEP) models examined baseline self-critical perfectionism, personal standards perfectionism, and demographic characteristics as predictors of profile membership while accounting for classification uncertainty. A sensitivity model additionally included baseline depressive symptoms.

The analysis was conducted within the Middle School Vulnerability Study. Zheng et al. (2024) used the same parent longitudinal dataset to examine whether repeated negative life events mediated associations between baseline neuroticism and repeated total and dimensional depressive symptoms using multilevel regression and mediation models. The present study instead represents a distinct secondary person-centered analysis of total Center for Epidemiologic Studies Depression Scale (CES-D) trajectories across six monthly follow-ups and tests perfectionism dimensions using growth mixture modeling and R3STEP. It is not presented as an independent replication.

### 2.2. Participants and Procedure

The study was conducted in two public secondary schools in Beijing, China, one serving an urban population and the other serving a rural population. Participants were recruited through the participating schools. At baseline (w0), 1150 adolescents aged 14 to 19 years participated (M = 16.27, SD = 0.91), including 576 females, 554 males, and 20 participants with missing sex information. Participants subsequently completed six monthly follow-up assessments (w1–w6). In this manuscript, T1–T6 refer to the six follow-up waves w1–w6.

Participation was voluntary, and responses were kept confidential. Written assent or consent, as applicable, was obtained from the adolescents, together with written consent from their parents or legal guardians before data collection. Ethical approval was granted by the Academic Ethics Review Committee of the College of Education, Capital Normal University (Approval No. CNU-CE-2022-05-2302; 23 May 2022). Participants received no financial or material incentives. The present secondary analysis used data collected under this approved longitudinal study protocol and involved no new participant contact or additional data collection.

All questionnaires were administered in Chinese during classroom-based, paper-and-pencil assessment sessions. Trained members of the research team provided standardized instructions and remained present throughout questionnaire completion. Teachers did not administer the survey, and students completed the questionnaires independently. Research staff checked returned questionnaires for obvious omissions, and questionnaires were collected immediately after completion. At baseline, participants completed demographic, perfectionism, and CES-D measures. The research team returned to the schools once per month for six consecutive months, and CES-D scores from w1 to w6 were used as trajectory indicators.

For trajectory enumeration, participants were required to have at least two non-missing CES-D follow-up assessments across w1–w6. This criterion yielded 1129 participants for the final trajectory model. Additional missingness and analytic sample information is reported in the Supplementary Materials.

### 2.3. Data Quality and Pre-Processing

The original survey did not include instructed-response attention-check items. Data quality was supported through standardized, supervised school-based administration, independent questionnaire completion, and immediate review of returned questionnaires for obvious omissions. Before the primary analyses, item-level data were screened for values outside permissible response ranges, coding inconsistencies, and scoring errors. Out-of-range entries were handled during the original pre-processing process before scale scores were computed. Positively worded CES-D items were reverse-scored according to the established scoring key. No additional participants were excluded using a post hoc careless-response criterion for the present analyses.

### 2.4. Measures

#### 2.4.1. Depressive Symptoms

Depressive symptoms were assessed using the 20-item Center for Epidemiological Studies Depression Scale (CES-D; Radloff, 1977). Items assess symptoms during the past week and are scored from 0 (rarely or none of the time) to 3 (most or all of the time). The four positively worded items were reverse-scored, and items were summed to create total scores ranging from 0 to 60, with higher scores indicating greater symptom severity. The established Chinese-language version was administered. CES-D scores from w1 to w6 were used as the six repeated trajectory indicators.

Baseline CES-D at w0 was not included as a trajectory indicator because the primary question concerned whether baseline perfectionism predicted subsequent symptom profiles. It was included in the sensitivity R3STEP model to examine associations beyond the initial symptom level. Internal consistency was high, with Cronbach’s α ranging from 0.911 to 0.945 across w1–w6; α for baseline CES-D was 0.889.

#### 2.4.2. Perfectionism Dimensions

Baseline perfectionism was assessed at w0 using the Dimensions of Perfectionism Scale for Children and Adolescents. The Chinese-language self-report measures used in the parent longitudinal project were developed using the translation and back-translation procedure described by Cohen et al. (2013). The original English-language measures were translated into Chinese by a bilingual translator from the Department of Psychology at Central South University and subsequently back-translated into English by another bilingual translator from the Department of Psychology at McGill University. Discrepancies identified through back-translation were resolved until agreement was reached. The present study used two seven-item dimensions: self-critical perfectionism (DPSCA4, 6, 7, 8, 10, 12, and 14) and personal standards perfectionism (DPSCA1, 2, 3, 5, 9, 11, and 13). For each dimension, scores were computed when at least six of seven items were available as the mean of valid items multiplied by seven. Higher scores indicate higher levels of the corresponding dimension. Raw scores were used for descriptive reporting, whereas standardized dimension scores were used in the R3STEP models; the same baseline standardization was retained across all prediction analyses. To avoid reproducing copyrighted scale content without explicit permission, the full Chinese and English item wording is not reproduced in the Supplementary Materials; item numbers, subscale allocation, scoring rules, and selected item-level psychometric diagnostics are provided instead. No separate documentation of an expert-panel review or pilot-testing phase was available in the retained project records; therefore, no such procedures are claimed here.

Dimension-specific reliability was evaluated on complete-item baseline samples. Self-critical perfectionism showed Cronbach’s α = 0.577, standardized α = 0.595, and McDonald’s ω = 0.607 (complete-case *N* = 1089). Personal standards perfectionism showed α = 0.637, standardized α = 0.637, and ω = 0.638 (complete-case *N* = 1110). Omega total was estimated from a one-factor principal-axis factor solution for each seven-item subscale using the complete-case Pearson correlation matrix. Item diagnostics identified DPSCA14 as the weakest self-critical item (corrected item-total correlation = 0.111), but no item was removed or rescored. The intended measurement structure was additionally evaluated with ordinal confirmatory factor analysis treating all 14 items as categorical and using WLSMV estimation. A correlated two-factor model was compared with a one-factor model without post hoc cross-loadings or residual correlations.

### 2.5. Predictors of Depressive Symptom Profile Membership

The primary R3STEP model simultaneously included standardized self-critical perfectionism, standardized personal standards perfectionism, age, sex, rural/urban source, and highest parental education. The demographic item recorded participants as female or male and was coded 0 = female and 1 = male; the variable is referred to as sex throughout the manuscript. Rural/urban source was coded 1 = rural and 2 = urban. Highest parental education was treated as an ordinal indicator. These variables were entered as predictors of latent profile membership after trajectory enumeration; they were not used to define or alter the latent profile solution. The sensitivity model additionally included baseline CES-D at w0.

### 2.6. Missing Data and Analytic Sample Flow

Missing data on repeated CES-D indicators were handled in Mplus using full-information maximum likelihood, allowing all available follow-up observations to contribute under a missing-at-random assumption conditional on observed data. Requiring at least two non-missing follow-up assessments yielded a trajectory enumeration sample of 1129. Automatic R3STEP applied listwise deletion to auxiliary predictor variables. Model 1 included 1033 participants and Model 2 included 1022 participants; the additional 11 exclusions in Model 2 were entirely attributable to missing baseline CES-D. Included-versus-excluded comparisons were conducted for focal predictors, demographic variables, baseline CES-D, follow-up availability, and descriptive most-likely trajectory membership. Because parental education accounted for most Model 1 exclusions (78 of 96 excluded cases), a targeted sensitivity analysis repeated both R3STEP models without parental education to evaluate whether this source of complete-case loss materially affected the focal perfectionism results.

### 2.7. Data Analysis

Descriptive statistics were computed for perfectionism dimensions, demographic predictors, baseline CES-D, and CES-D scores across the six monthly follow-ups. Continuous variables were summarized using means and standard deviations, and categorical variables using frequencies and percentages.

Growth mixture modeling was used to identify heterogeneous profiles of CES-D scores across w1–w6. Trajectory enumeration, full-information maximum likelihood estimation, R3STEP models, and the baseline CES-D sensitivity model were conducted in Mplus Version 8 (Muthén & Muthén, 1998–2017). Stata Version 15 was used for data checking, descriptive summaries, and figure preparation (StataCorp, 2017).

The longitudinal growth form was re-evaluated before mixture modeling. A quadratic single-class growth model fit better than the linear alternative and yielded a significant quadratic mean and variance; the quadratic form was therefore retained for all subsequent mixture models. The final CES-D trajectory model was a three-class quadratic FULL growth mixture model with intercept, linear slope, and quadratic slope factors. Time scores were fixed at 0, 1, 2, 3, 4, and 5 for w1–w6. Growth-factor means varied across classes, whereas growth-factor variances and covariances and wave-specific residual variances were estimated and constrained equal across classes. These equality constraints were used to limit class-specific covariance complexity and support stable, interpretable class enumeration, particularly given the relatively small non-majority classes; alternative variance specifications were examined as robustness checks. The final solution terminated normally, replicated the best log-likelihood, and showed no active non-positive definite PSI warning.

Robustness checks included quadratic latent class growth analysis (LCGA) solutions from one through five classes with formal bootstrap likelihood ratio testing. In addition, targeted variance-specification checks were conducted around the lower-class boundary: a two-class FULL GMM was inadmissible because of PSI/quadratic-factor problems, whereas diagonal and random-intercept/random-slope with fixed quadratic-variance alternatives were admissible; the three-class FULL GMM was admissible and reproduced the established three-profile structure. A relaxed trajectory-eligibility sensitivity sample requiring at least one of six follow-up CES-D assessments (*N* = 1146) also reproduced the three retained profiles closely.

After selection of the three-profile solution, automatic R3STEP estimated predictors of profile membership while preserving the selected solution and accounting for classification uncertainty (Asparouhov & Muthén, 2014). The low-stable profile was the reference. Model 1 included both perfectionism dimensions and the four demographic predictors; Model 2 additionally included baseline CES-D. To directly compare the self-critical and personal-standards coefficients within each class contrast, a manual three-step implementation was constructed solely for coefficient-contrast testing. It reproduced the automatic R3STEP focal coefficients and standard errors to within 0.001 and 0.000, respectively, and exact parameter covariances were then used for Wald tests of the self-critical minus personal-standards coefficient difference. The automatic R3STEP estimates remain the primary prediction results.

Observed class-specific CES-D means were summarized by most likely profile membership. School and classroom identifiers were unavailable, so cluster-adjusted or multilevel analyses could not be estimated; rural/urban source was included as a contextual predictor but was confounded with the two participating schools.

## 3. Results

### 3.1. Descriptive Statistics

The trajectory enumeration sample included 1129 adolescents with at least two non-missing CES-D follow-up assessments. Mean age was 16.26 years (SD = 0.90). Mean raw scores were 14.10 (SD = 2.72) for self-critical perfectionism and 19.59 (SD = 2.91) for personal standards perfectionism. Mean CES-D scores across the six monthly follow-ups ranged from 11.81 to 13.17. Selected descriptive statistics and coding information are reported in the Supplementary Materials.

### 3.2. DPSCA Psychometric Checks

For self-critical perfectionism, internal consistency was modest (α = 0.577; standardized α = 0.595; ω = 0.607). DPSCA14 had the lowest corrected item-total correlation (0.111), and deleting that item would have increased α by approximately 0.032; however, the original seven-item subscale composition was retained unchanged. Personal standards perfectionism showed α = 0.637, standardized α = 0.637, and ω = 0.638, with corrected item-total correlations ranging from 0.273 to 0.424 and no item whose deletion materially increased α. The analysis scores were reproduced exactly from the item-level source data under the ≥6/7 scoring rule.

The one-factor ordinal CFA fit poorly, χ^2^(77) = 1553.452, *p* < 0.001, CFI = 0.445, TLI = 0.344, RMSEA = 0.130, 90% CI [0.124, 0.135], SRMR = 0.108. The correlated two-factor model fit substantially better, χ^2^(76) = 860.809, *p* < 0.001, CFI = 0.705, TLI = 0.647, RMSEA = 0.095, 90% CI [0.089, 0.101], SRMR = 0.079; WLSMV DIFFTEST χ^2^(1) = 214.616, *p* < 0.001. The latent correlation between self-critical and personal standards perfectionism was 0.166 (SE = 0.044, *p* < 0.001). The two-factor model nevertheless showed limited absolute fit, and DPSCA14 had a particularly low standardized loading (0.210; R^2^ = 0.044).

### 3.3. Latent Depressive Symptom Profile Model Selection

The retained three-class quadratic FULL GMM used 1129 adolescents and yielded log-likelihood = −21,131.809, AIC = 42,309.619, BIC = 42,425.288, sample-size adjusted BIC = 42,352.233, and entropy = 0.834. The solution terminated normally, replicated the best log-likelihood, and showed no active PSI warning. Quadratic LCGA robustness models from one through five classes showed monotonically improving information criteria and significant BLRT results through five classes, but the LCGA solutions increasingly divided participants into severity strata rather than reproducing the distinct decreasing and increasing longitudinal symptom course shapes captured by the three-class FULL GMM. The targeted variance-specification checks further showed that the two-class FULL GMM was inadmissible, whereas the three-class FULL GMM was admissible and reproduced the established structure. On the combined statistical, stability, parsimony, and substantive evidence, the three-class FULL GMM was retained as the final trajectory solution. The results are presented in Table 1.

### 3.4. Characteristics of the Final Symptom Profiles

**Figure 1:**
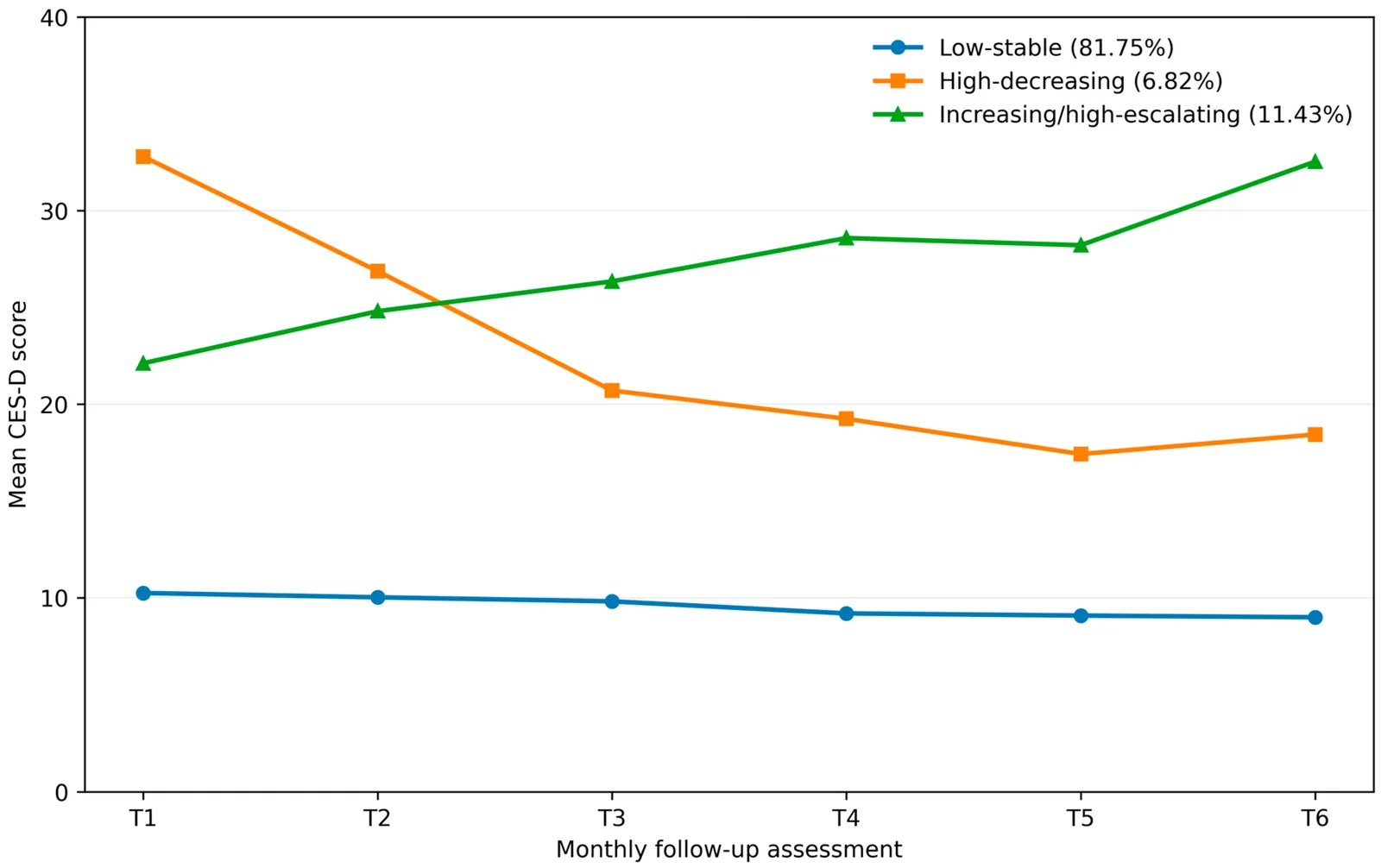
Observed mean CES-D scores across six monthly follow-up assessments by most likely depressive symptom profile. Note. Figure 1 presents observed w1–w6 means calculated by most likely class membership from the retained primary trajectory model. Class 1 = low-stable; Class 2 = high-decreasing; Class 3 = increasing/high-escalating. CES-D scores range from 0 to 60.

The retained model identified low-stable, high-decreasing, and increasing/high-escalating profiles across the six monthly follow-ups (Figure 1; Table 2 and Table 3). The profiles are sample- and model-dependent symptom patterns rather than clinical diagnoses. The low-stable profile included 923 adolescents (81.75%) and remained consistently low, declining from 10.24 at T1 to 8.99 at T6. The high-decreasing profile included 77 adolescents (6.82%), beginning at 32.79 and declining to 18.43. The increasing/high-escalating profile included 129 adolescents (11.43%) and rose from 22.11 to 32.53.

### 3.5. Perfectionism and Demographic Predictors of Profile Membership

Table 4 reports all predictors from Model 1 and Model 2. In Model 1 (*N* = 1033), a 1-SD increase in self-critical perfectionism was associated with higher odds of high-decreasing membership (OR = 3.47, 95% CI [2.48, 4.87], *p* < 0.001) and increasing/high-escalating membership (OR = 3.13, 95% CI [2.32, 4.23], *p* < 0.001) relative to low-stable membership. Personal standards perfectionism was not significant for either comparison (high-decreasing: OR = 0.77, 95% CI [0.57, 1.02], *p* = 0.072; increasing/high-escalating: OR = 0.90, 95% CI [0.65, 1.26], *p* = 0.549). No demographic predictor significantly differentiated high-decreasing from low-stable membership. For increasing/high-escalating versus low-stable membership, older age (OR = 1.54, 95% CI [1.12, 2.12], *p* = 0.008), female rather than male sex (male vs. female: OR = 0.39, 95% CI [0.21, 0.70], *p* = 0.002), and higher parental education (OR = 1.62, 95% CI [1.13, 2.31], *p* = 0.008) were associated with profile membership.

### 3.6. Baseline CES-D Adjustment, Direct Coefficient Contrasts, and Sensitivity Analyses

In Model 2 (*N* = 1022), self-critical perfectionism remained associated with high-decreasing (OR = 1.92, 95% CI [1.30, 2.81], *p* = 0.001) and increasing/high-escalating membership (OR = 1.79, 95% CI [1.28, 2.49], *p* = 0.001), although estimates were attenuated. Personal standards perfectionism remained non-significant. Baseline CES-D strongly differentiated both high-decreasing (OR = 1.26 per score point, 95% CI [1.18, 1.33], *p* < 0.001) and increasing/high-escalating membership (OR = 1.25, 95% CI [1.18, 1.32], *p* < 0.001). For increasing/high-escalating membership, older age (OR = 1.74, 95% CI [1.10, 2.74], *p* = 0.017) and higher parental education (OR = 1.72, 95% CI [1.07, 2.76], *p* = 0.025) remained significant; the sex estimate was attenuated to *p* = 0.052.

Direct Wald contrasts showed that in Model 1 the self-critical coefficient was significantly larger than the personal-standards coefficient for both high-decreasing versus low-stable (difference = 1.512, SE = 0.240, Wald χ^2^(1) = 39.740, *p* < 0.001, 95% CI [1.042, 1.982]) and increasing/high-escalating versus low-stable (difference = 1.241, SE = 0.250, Wald χ^2^(1) = 24.711, *p* < 0.001, 95% CI [0.752, 1.730]). After baseline CES-D adjustment, however, the corresponding differences were not significant (high-decreasing: difference = 0.545, SE = 0.301, *p* = 0.070; increasing/high-escalating: difference = 0.217, SE = 0.303, *p* = 0.474). Thus, evidence that self-critical perfectionism had a stronger coefficient than personal standards was confined to the primary model.

Included-versus-excluded comparisons showed negligible differences for self-critical perfectionism, personal standards perfectionism, baseline CES-D, age, sex, rural/urban source, and the descriptive most-likely trajectory distribution. The main source of R3STEP exclusion was missing parental education. Accordingly, targeted sensitivity models omitted parental education, increasing the analytic samples to 1100 and 1088 for Models 1 and 2, respectively. All four self-critical effects retained their direction and statistical significance, with a maximum absolute coefficient change of 0.072. Personal-standards estimates were also directionally stable; one borderline Model 1 estimate for high-decreasing membership shifted from *p* = 0.072 to *p* = 0.049 while changing only modestly in magnitude (B = −0.267 to −0.298). This pattern did not alter the conclusion that personal standards showed no consistent association across models.

A second sensitivity analysis addressed the weak psychometric performance of DPSCA14 by recalculating self-critical perfectionism from the remaining six items (DPSCA4, 6, 7, 8, 10, and 12; scored when at least five of six items were available). The four self-critical R3STEP associations retained the same directions and statistical significance status as in the primary seven-item analysis. The maximum absolute coefficient change was 0.068 (maximum relative change = 10.46%), and the analytic sample sizes were unchanged (Model 1 *N* = 1033; Model 2 *N* = 1022). Thus, exclusion of DPSCA14 did not materially alter the substantive self-critical perfectionism findings (Table S10).

## 4. Discussion

### 4.1. Principal Findings

Across six monthly follow-ups, three depressive symptom profiles were identified: low-stable, high-decreasing, and increasing/high-escalating. Self-critical perfectionism differentiated both non-low-stable profiles from the low-stable profile when personal standards and demographic characteristics were entered simultaneously. These associations remained after baseline depressive symptoms were included, although they were attenuated. Personal standards perfectionism showed no consistent association. Direct coefficient contrasts showed stronger self-critical than personal-standards effects in the primary model, but these differences were not significant after baseline CES-D adjustment. Baseline depressive symptoms strongly predicted both non-low-stable profiles. Among secondary demographic predictors, older age, female sex, and higher parental education were associated with increasing/high-escalating membership in Model 1; age and parental education remained significant after baseline symptom adjustment, whereas the sex estimate was attenuated.

### 4.2. Depressive Symptom Profiles and Perfectionism

The predominance of a low-stable profile alongside smaller elevated profiles is consistent with person-centered studies of adolescent depressive symptoms (Batterham et al., 2026; Jamil et al., 2025; Pakhomova et al., 2026; Reck et al., 2024). The profiles should be interpreted as sample- and model-dependent summaries, not diagnostic categories. The high-decreasing group began with the highest symptoms and improved, whereas the increasing/high-escalating group worsened across the short observation period.

The self-critical pattern is consistent with multidimensional perfectionism theories that distinguish evaluative concerns from high standards or striving (Dunkley et al., 2006; Hewitt & Flett, 1991; Stoeber & Otto, 2006). Harsh self-evaluation, concern over mistakes, and contingent self-worth align more closely with depressive vulnerability than demanding goals alone (Limburg et al., 2017; Smith et al., 2020, 2021). The findings provide a person-centered extension of this established association: self-critical perfectionism differentiated adolescents with elevated symptom liability from those who remained low.

The direct coefficient tests refine this interpretation. Before baseline CES-D adjustment, self-critical perfectionism had significantly larger coefficients than personal standards perfectionism for both non-low-stable profiles. After baseline CES-D was added, self-critical perfectionism remained independently associated with both profiles, but the direct self-critical-versus-personal-standards differences were no longer significant. The results therefore support the greater consistency of self-critical perfectionism while cautioning against treating its relative advantage over personal standards as invariant to baseline symptom burden. Because self-critical perfectionism predicted both high-decreasing and increasing/high-escalating membership, it should not be described as an escalation-specific marker.

Attenuation after baseline CES-D adjustment indicates that self-critical perfectionism shared substantial variance with initial symptom burden but retained incremental information. This is compatible with reciprocal longitudinal relations between perfectionism and depressive symptoms (Smith et al., 2021). Recent longitudinal evidence in young women similarly found reciprocal relations between perfectionistic concerns and depressive symptoms, whereas perfectionistic strivings did not predict subsequent depressive symptoms (Zotschew et al., 2026). Baseline adjustment cannot fully resolve state-trait overlap and may remove part of a pathway through which vulnerability is expressed. The results therefore do not establish a causal effect of self-critical perfectionism on later profile membership.

### 4.3. Demographic Predictors

The demographic findings were secondary and should be interpreted cautiously. Older age and higher parental education were associated with increasing/high-escalating membership in both models, whereas the female–male difference in Model 1 was attenuated after baseline symptoms were entered. None of the demographic predictors differentiated high-decreasing from low-stable membership. These patterns were secondary and exploratory and should not be interpreted as confirmatory findings; the study cannot determine whether they generalize beyond the participating schools. Rural/urban source was also confounded with school because one school served each population.

### 4.4. Implications

The findings support treating perfectionism as multidimensional in personality vulnerability research. The relevant signal was not high standards alone but the self-critical meaning attached to mistakes and perceived failure. For school-based research, this pattern motivates further study of flexible self-evaluation and responses to setbacks rather than attempts to reduce ambition or standards. The present observational analysis did not test screening, prevention, or intervention effects; self-critical perfectionism should therefore be regarded as a probabilistic group-level marker, not a stand-alone tool for identifying individuals or a demonstrated treatment target.

### 4.5. Strengths and Limitations

Strengths include six repeated symptom assessments, a sensitivity-checked three-class growth mixture solution, R3STEP adjustment for classification uncertainty, simultaneous estimation of two perfectionism dimensions and demographic predictors, formal direct coefficient contrasts validated against automatic R3STEP, and targeted selection-sensitivity analyses.

Several limitations qualify the findings. The design was observational and relied on self-report. The original survey had no instructed-response attention checks, although administration was standardized and data-range, coding, and scoring checks were conducted. Participants came from only two public secondary schools, one urban and one rural; school and classroom identifiers were unavailable, so clustering could not be modeled and school was confounded with rural/urban source. Consequently, standard errors may not fully account for within-school or within-class dependence. The observation period was approximately six months, the high-decreasing profile was small, and ages ranged from 14 to 19 years. The six-month window cannot establish whether the increasing profile would continue to worsen, stabilize, or subsequently decline, nor whether the high-decreasing profile reflects a durable developmental course rather than short-term recovery from an initially elevated symptom state. The study also did not assess digitally mediated peer-comparison or identity processes; recent work in early adolescents highlights social-media-related identity and information-environment processes as potentially relevant contextual influences (De Lorenzo et al., 2026). Automatic R3STEP used listwise deletion for predictors, although included-versus-excluded comparisons showed little evidence of systematic differences in the focal perfectionism variables and the main source of exclusion was parental education. Removing parental education in a targeted sensitivity analysis did not materially alter the self-critical findings. Most importantly, the DPSCA subscales showed only modest internal consistency in this sample, particularly self-critical perfectionism, and although a correlated two-factor ordinal CFA fit substantially better than a one-factor model, its absolute fit remained limited and DPSCA14 had a weak loading. These measurement limitations may attenuate or destabilize associations and warrant replication with stronger subscale measurement. Perfectionism was assessed only at baseline and only two dimensions were examined.

## 5. Conclusions

Across six monthly follow-ups, self-critical perfectionism differentiated adolescents in both non-low-stable depressive symptom profiles from those in the low-stable profile, including after baseline depressive symptoms were considered. Personal standards perfectionism showed no consistent association. Direct coefficient contrasts indicated stronger self-critical than personal-standards effects in the primary model, but these differences were not retained after baseline CES-D adjustment. The findings therefore support self-critical perfectionism as a comparatively consistent probabilistic marker of elevated symptom liability while underscoring the need for caution because the DPSCA subscales showed modest reliability and limited absolute factorial fit. Causal, screening, and intervention interpretations are not warranted.

## Figures and Tables

**Table 1:** Fit and robustness evidence for the retained CES-D trajectory solution.

Specification	K	LL	AIC	BIC	aBIC	Entropy	BLRT *p*	Status
FULL GMM	3	−21,131.809	42,309.619	42,425.288	42,352.233	0.834	—	Retained
LCGA	1	−23,846.727	47,711.454	47,756.716	47,728.129	—	—	Robustness
LCGA	2	−22,186.258	44,398.516	44,463.894	44,422.602	0.922	<0.001	Robustness
LCGA	3	−21,638.166	43,310.331	43,395.826	43,341.829	0.895	<0.001	Robustness
LCGA	4	−21,400.060	42,842.120	42,947.731	42,881.029	0.886	<0.001	Robustness
LCGA	5	−21,314.014	42,678.028	42,803.755	42,724.348	0.849	<0.001	Robustness

Note. BLRT = bootstrap likelihood ratio test; LCGA = latent class growth analysis; FULL GMM = growth mixture model with shared growth-factor variances/covariances estimated across classes. All LCGA K = 2–5 BLRT runs completed 500/500 bootstrap draws and reproduced the corresponding K-1 H0 log-likelihood. The FULL GMM K = 2 specification was inadmissible because of PSI/quadratic-factor problems; the FULL GMM K = 3 specification was admissible.

**Table 2:** Class size, classification quality, and observed CES-D scores by retained profile.

Class	Profile	N	%	APP	T1	T2	T3	T4	T5	T6
1	Low-stable	923	81.75	0.950	10.24	10.02	9.81	9.19	9.08	8.99
2	High-decreasing	77	6.82	0.854	32.79	26.87	20.70	19.24	17.42	18.43
3	Increasing/high-escalating	129	11.43	0.809	22.11	24.80	26.34	28.58	28.21	32.53

Note. T1–T6 are observed means by most likely profile membership. The low-stable profile (original Class 1) was the R3STEP reference.

**Table 3:** Growth-factor means for the retained three-class quadratic FULL GMM.

Profile	Parameter	Estimate	SE	*p*	95% CI
Low-stable	Intercept	10.230	0.448	<0.001	[9.352, 11.107]
Low-stable	Linear slope	−0.323	0.235	0.170	[−0.783, 0.138]
Low-stable	Quadratic slope	0.013	0.044	0.775	[−0.074, 0.099]
High-decreasing	Intercept	31.173	1.574	<0.001	[28.089, 34.258]
High-decreasing	Linear slope	−6.799	1.415	<0.001	[−9.571, −4.026]
High-decreasing	Quadratic slope	0.822	0.260	0.002	[0.312, 1.331]
Increasing/high-escalating	Intercept	21.749	1.310	<0.001	[19.182, 24.315]
Increasing/high-escalating	Linear slope	1.584	0.917	0.084	[−0.213, 3.381]
Increasing/high-escalating	Quadratic slope	0.041	0.229	0.859	[−0.408, 0.489]

Note. Growth-factor variances and covariances were shared across classes. The observed increasing profile should not be interpreted as having individually significant positive linear or quadratic growth-factor means, because its slope parameters did not reach conventional significance.

**Table 4:** Perfectionism and demographic predictors of depressive symptom profile membership.

Comparison	Predictor	Model 1 OR [95% CI]; *p*	Model 2 OR [95% CI]; *p*
High-decreasing vs. low-stable	Self-critical perfectionism (z)	3.47 [2.48, 4.87]; <0.001	1.92 [1.30, 2.81]; 0.001
High-decreasing vs. low-stable	Personal standards perfectionism (z)	0.77 [0.57, 1.02]; 0.072	1.11 [0.74, 1.66]; 0.608
High-decreasing vs. low-stable	Age	1.16 [0.83, 1.62]; 0.386	1.24 [0.83, 1.84]; 0.288
High-decreasing vs. low-stable	Sex: male vs. female	0.79 [0.42, 1.51]; 0.481	1.00 [0.47, 2.13]; 0.990
High-decreasing vs. low-stable	Urban vs. rural source	1.20 [0.64, 2.25]; 0.566	1.67 [0.75, 3.72]; 0.206
High-decreasing vs. low-stable	Highest parental education	1.15 [0.85, 1.55]; 0.380	1.16 [0.80, 1.69]; 0.426
High-decreasing vs. low-stable	Baseline CES-D	—	1.26 [1.18, 1.33]; <0.001
Increasing/high-escalating vs. low-stable	Self-critical perfectionism (z)	3.13 [2.32, 4.23]; <0.001	1.79 [1.28, 2.49]; 0.001
Increasing/high-escalating vs. low-stable	Personal standards perfectionism (z)	0.90 [0.65, 1.26]; 0.549	1.44 [0.89, 2.31]; 0.134
Increasing/high-escalating vs. low-stable	Age	1.54 [1.12, 2.12]; 0.008	1.74 [1.10, 2.74]; 0.017
Increasing/high-escalating vs. low-stable	Sex: male vs. female	0.39 [0.21, 0.70]; 0.002	0.47 [0.22, 1.01]; 0.052
Increasing/high-escalating vs. low-stable	Urban vs. rural source	0.67 [0.37, 1.22]; 0.192	1.14 [0.52, 2.53]; 0.741
Increasing/high-escalating vs. low-stable	Highest parental education	1.62 [1.13, 2.31]; 0.008	1.72 [1.07, 2.76]; 0.025
Increasing/high-escalating vs. low-stable	Baseline CES-D	—	1.25 [1.18, 1.32]; <0.001

Note. Reference profile = low-stable. Model 1 simultaneously included both perfectionism dimensions, age, sex, rural/urban source, and highest parental education (*N* = 1033). Model 2 additionally included baseline CES-D (*N* = 1022). Sex was coded 0 = female, 1 = male; rural/urban source was coded 1 = rural, 2 = urban. Perfectionism predictors were standardized baseline scores; age, parental education, and baseline CES-D were entered in their original units. *p* values derive from the logistic coefficient tests.

## Data Availability

The data are not publicly available because they contain sensitive information from adolescent participants and are subject to institutional and ethical restrictions. Requests for access to de-identified data may be directed to the corresponding author and will be considered subject to applicable ethical and institutional approvals. Analysis code and materials may also be made available where permitted.

## Supplementary Materials

The following supporting information can be downloaded at: https://www.mdpi.com/article/10.3390/bs16091678/s1, Table S1, selected descriptive statistics, scoring, and coding information; Table S2A–D, analytic sample flow, included-versus-excluded comparisons, and predictor missingness decomposition; Table S3, DPSCA subscale psychometrics and selected item diagnostics; Table S4A,B, ordinal CFA model comparison and selected diagnostics; Table S5A,B, CES-D trajectory model fit and robustness evidence; Table S6, class-specific growth-factor estimates; Table S7, full R3STEP results; Table S8, direct self-critical-versus-personal-standards coefficient contrasts; Table S9, targeted parent-education exclusion sensitivity analyses; Table S10, sensitivity analysis excluding DPSCA14 from the self-critical perfectionism score; and Figure S1, perfectionism dimensions predicting depressive symptom profile membership in Model 1 and Model 2. To avoid reproducing copyrighted scale content without explicit permission, the full Chinese and English item wording is not reproduced; item numbers, subscale allocation, scoring rules, and selected psychometric diagnostics are reported instead.

## References

[B1-behavsci-16-01678] Asparouhov T., Muthén B. (2014). Auxiliary variables in mixture modeling: Three-step approaches using Mplus. Structural Equation Modeling: A Multidisciplinary Journal.

[B2-behavsci-16-01678] Batterham P. J., Maston K., O’Dea B., Brown L., Calear A. L., Larsen M., Skinner S. R., Christensen H., Werner-Seidler A. (2026). Stress-diathesis based predictors of depression and anxiety trajectories in adolescence: A population-based longitudinal cohort study. Psychological Medicine.

[B3-behavsci-16-01678] Cohen J. R., Hankin B. L., Gibb B. E., Hammen C., Hazel N. A., Ma D., Yao S., Zhu X. Z., Abela J. R. Z. (2013). Negative attachment cognitions and emotional distress in mainland Chinese adolescents: A prospective multi-wave test of vulnerability-stress and stress generation models. Journal of Clinical Child & Adolescent Psychology.

[B4-behavsci-16-01678] De Lorenzo A., Di Gesto C., Graziano F., Calandri E., Kaakinen M., Oksanen A., Rabaglietti E. (2026). Psychometric properties of the Italian version of the 6-Item Identity Bubble Reinforcement Scale (IBRS-6). Humanities and Social Sciences Communications.

[B5-behavsci-16-01678] Dunkley D. M., Blankstein K. R., Zuroff D. C., Lecce S., Hui D. (2006). Self-critical and personal standards factors of perfectionism located within the five-factor model of personality. Personality and Individual Differences.

[B6-behavsci-16-01678] Flett G. L., Hewitt P. L., Besser A., Su C., Vaillancourt T., Boucher D., Munro Y., Davidson L. A., Gale O. (2016). The child–adolescent perfectionism scale: Development, psychometric properties, and associations with stress, distress, and psychiatric symptoms. Journal of Psychoeducational Assessment.

[B7-behavsci-16-01678] Hewitt P. L., Flett G. L. (1991). Perfectionism in the self and social contexts: Conceptualization, assessment, and association with psychopathology. Journal of Personality and Social Psychology.

[B8-behavsci-16-01678] Jamil B., Su J., Elam K. K., Lemery-Chalfant K., Cruz R., Grimm K. J., Seaton E. K. (2025). Polygenic risk and trajectories of depressive symptoms in diverse adolescents: Gene–environment interplay with family conflict and parental acceptance. Development and Psychopathology.

[B9-behavsci-16-01678] Limburg K., Watson H. J., Hagger M. S., Egan S. J. (2017). The relationship between perfectionism and psychopathology: A meta-analysis. Journal of Clinical Psychology.

[B10-behavsci-16-01678] Lunn J., Greene D., Callaghan T., Egan S. J. (2023). Associations between perfectionism and symptoms of anxiety, obsessive-compulsive disorder and depression in young people: A meta-analysis. Cognitive Behaviour Therapy.

[B11-behavsci-16-01678] Muthén L. K., Muthén B. O. (1998–2017). Mplus user’s guide.

[B12-behavsci-16-01678] Pakhomova T. E., Beksinska M., Dietrich J. J., Salway T. J., Gadermann A., Closson K., Rowlands A., Jesson J., Ndung’u T., Brockman M., Nduna M., Vermaak S., Smit J., Kaida A. (2026). Twelve-month trajectories of depressive symptoms and associated socio-structural factors among adolescents and young adults in Soweto and Durban, South Africa. Journal of Affective Disorders.

[B13-behavsci-16-01678] Radloff L. S. (1977). The CES-D scale: A self-report depression scale for research in the general population. Applied Psychological Measurement.

[B14-behavsci-16-01678] Reck A., Seaton E. K., Oshri A., Kogan S. M. (2024). Trajectories of depressive symptoms among Black American adolescents: Sociocultural, racism and familial predictors. Journal of Clinical Child & Adolescent Psychology.

[B15-behavsci-16-01678] Smith M. M., Sherry S. B., Ray C., Hewitt P. L., Flett G. L. (2021). Is perfectionism a vulnerability factor for depressive symptoms, a complication of depressive symptoms, or both? A meta-analytic test of 67 longitudinal studies. Clinical Psychology Review.

[B16-behavsci-16-01678] Smith M. M., Sherry S. B., Vidovic V., Hewitt P. L., Flett G. L. (2020). Why does perfectionism confer risk for depressive symptoms? A meta-analytic test of the mediating role of stress and social disconnection. Journal of Research in Personality.

[B17-behavsci-16-01678] StataCorp (2017). Stata statistical software: Release 15 *[Computer software]*.

[B18-behavsci-16-01678] Stoeber J., Otto K. (2006). Positive conceptions of perfectionism: Approaches, evidence, challenges. Personality and Social Psychology Review.

[B19-behavsci-16-01678] Thapar A., Collishaw S., Pine D. S., Thapar A. K. (2012). Depression in adolescence. The Lancet.

[B20-behavsci-16-01678] Vaillancourt T., Haltigan J. D. (2018). Joint trajectories of depression and perfectionism across adolescence and childhood risk factors. Development and Psychopathology.

[B21-behavsci-16-01678] Zheng S., Wang J., Lu S., Xiao J. (2024). A longitudinal investigation of the cross-dimensional mediating role of negative life events between neuroticism and depressive symptoms in adolescents. Journal of Affective Disorders.

[B22-behavsci-16-01678] Zotschew T., Claus N., Egan S. J., Shafran R., Ehring T., Takano K., Limburg K., Cludius B. (2026). Perfectionism as a transdiagnostic process in depression and generalized anxiety disorder: Evidence from a longitudinal study of young women. Journal of Anxiety Disorders.

