# Supplementary material for: Perfectionism Dimensions and Longitudinal Depressive Symptom Profiles in Adolescents: Distinguishing Evaluative Self-Criticism from Personal Standards
Source: Behav Sci (Basel). 2026 Sep 17;16(9):1678. doi: 10.3390/bs16091678 (PMC13603892; doi:10.3390/bs16091678)
Supplement: Supplementary file 1 [file behavsci-16-01678-s001.zip › behavsci-4515163-supplementary.pdf]

Supplementary Materials

Perfectionism Dimensions and Longitudinal Depressive Symptom Profiles in Adolescents: Distinguishing Evaluative Self-Criticism from Personal Standards

Supplementary note on DPSCA translation and copyright.

The Chinese-language self-report measures used in the parent longitudinal project were developed using translation and back-translation. The original English-language measures were translated into Chinese by a bilingual translator from the Department of Psychology at Central South University and back-translated into English by another bilingual translator from the Department of Psychology at McGill University. Discrepancies were resolved until agreement was reached. To avoid reproducing copyrighted scale content without explicit permission, the full Chinese and English item wording is not reproduced here; item numbers, subscale allocation, scoring rules, and selected item-level psychometric diagnostics are reported instead.

Table S1. Selected descriptive statistics, scoring, and coding information.

Variable / feature	Value / coding	Analytic note
Baseline sample size	1150	Participants aged 14–19 years
Baseline age, M (SD)	16.27 (0.91)	Full baseline sample
Sex	576 female; 554 male; 20 missing	Coded 0=female, 1=male in prediction models
Trajectory sample age, M (SD)	16.26 (0.90)	N=1129 trajectory-eligible sample
Self-critical perfectionism, raw M (SD)	14.10 (2.72)	Seven-item subscale; higher=more self-critical perfectionism
Personal standards perfectionism, raw M (SD)	19.59 (2.91)	Seven-item subscale; higher=more personal standards perfectionism
Self-critical items	DPSCA4, 6, 7, 8, 10, 12, 14	≥6/7 valid items; mean(valid)×7
Personal standards items	DPSCA1, 2, 3, 5, 9, 11, 13	≥6/7 valid items; mean(valid)×7
Perfectionism model scaling	Baseline z scores	Raw scores used descriptively; z scores used in R3STEP
Rural/urban source	1=rural; 2=urban	School and rural/urban source were confounded
Highest parental education	Ordinal	Entered in original units
CES-D trajectory indicators	w1–w6; 0–60	T1–T6 in manuscript; follow-up means ranged 11.81–13.17

Note. Perfectionism scoring used the original seven-item subscale compositions. Full DPSCA item wording is not reproduced because explicit reproduction permission was not available.

Table S2A. Analytic sample flow.

Stage	Included N	Excluded N	Reason / rule
Trajectory eligible	1129	—	≥2 nonmissing w1–w6 CES-D assessments
Model 1	1033	96	Automatic R3STEP listwise deletion on six auxiliary predictors
Model 2	1022	107	Adds baseline CES-D
Additional exclusions in Model 2	11	—	All 11 had missing baseline CES-D

Table S2B. Included-versus-excluded continuous-variable comparisons.

Variable	M1 SMD	Magnitude	Welch p	M2 SMD	Magnitude	Welch p
Self-critical z	.046	Negligible	.711	.005	Negligible	.966
Personal standards z	-.070	Negligible	.504	-.007	Negligible	.948

Variable	M1 SMD	Magnitude	Welch p	M2 SMD	Magnitude	Welch p
Baseline CES-D	.033	Negligible	.756	.033	Negligible	.756
Age	.037	Negligible	.755	.071	Negligible	.508
Parent education	.554	Large	.033	.351	Noticeable	.059
Follow-up valid count	.144	Small	.260	.142	Small	.232

Note. SMD = standardized mean difference. Parent education was the principal source of complete-case loss.

Table S2C. Included-versus-excluded categorical-variable comparisons.

Variable	M1 Cramer's V	M1 p	M2 Cramer's V	M2 p
Gender	.044	.143	.045	.131
Area/source	.032	.285	.038	.202
Most-likely trajectory class	.041	.386	.020	.790

Note. Most-likely class comparisons are descriptive selection checks only.

Table S2D. Predictor missingness decomposition among excluded participants.

Variable	Model 1 excluded: missing N (%)	Model 2 excluded: missing N (%)
Self-critical perfectionism z score	11 (11.5%)	11 (10.3%)
Personal standards perfectionism z score	11 (11.5%)	11 (10.3%)
Sex	19 (19.8%)	19 (17.8%)
Age	17 (17.7%)	17 (15.9%)
Rural/urban source	0 (0.0%)	0 (0.0%)
Highest parental education	78 (81.2%)	78 (72.9%)
Baseline CES-D	Not included in Model 1	17 (15.9%)

Note. Percentages use each model's excluded sample as the denominator (Model 1 N=96; Model 2 N=107). Missingness counts can overlap because an excluded participant could be missing more than one auxiliary variable.

Table S3. DPSCA subscale psychometrics and selected item diagnostics.

Subscale	Complete-case N	≥6/7 eligible N	Raw α	Std. α	ω total	Min item-total r	Max item-total r	α-if-deleted flag
Self-critical perfectionism	1089	1139	.577	.595	.607	.111 (DPSCA14)	.409	DPSCA14: +.032
Personal standards perfectionism	1110	1139	.637	.637	.638	.273	.424	No increase ≥.02

Note. No item was reversed, deleted, rescored, or reassigned. DPSCA14 was retained despite the weakest corrected item-total correlation.

Table S4A. DPSCA ordinal CFA model comparison.

Model	N	χ² (df)	p	CFI	TLI	RMSEA [90% CI]	SRMR
One-factor	1143	1553.452 (77)	<.001	.445	.344	.130 [.124, .135]	.108
Correlated two-factor	1143	860.809 (76)	<.001	.705	.647	.095 [.089, .101]	.079

Note. WLSMV estimation with DPSCA1–DPSCA14 declared categorical. DIFFTEST χ²(1)=214.616, p<.001, favoring the correlated two-factor model.

Table S4B. Selected two-factor CFA diagnostics.

Parameter	Estimate	SE	p	95% CI	Diagnostic note
Latent factor correlation	.166	.044	<.001	[.080, .251]	Self-critical WITH personal standards
DPSCA14 loading	.210	.036	<.001	[.139, .281]	R^2=.044; low loading
DPSCA4 loading	.364	—	—	—	Modest loading
DPSCA10 loading	.352	—	—	—	Modest loading

Note. All other two-factor loadings had no loading-quality flag; no negative loading, Heywood case, factor correlation ≥.95, or other improper solution was observed.

Table S5A. CES-D trajectory model fit and robustness evidence.

Specification	K	LL	AIC	BIC	aBIC	Entropy	BLRT p	Status
FULL GMM	3	-21131.809	42309.619	42425.288	42352.233	.834	—	Retained
LCGA	1	-23846.727	47711.454	47756.716	47728.129	—	—	Robustness
LCGA	2	-22186.258	44398.516	44463.894	44422.602	.922	<.001	Robustness
LCGA	3	-21638.166	43310.331	43395.826	43341.829	.895	<.001	Robustness
LCGA	4	-21400.060	42842.120	42947.731	42881.029	.886	<.001	Robustness
LCGA	5	-21314.014	42678.028	42803.755	42724.348	.849	<.001	Robustness

Note. The retained three-class trajectory solution was not re-estimated or reordered. The two-class FULL GMM was inadmissible in targeted variance-specification checks, whereas the three-class FULL GMM was admissible. Subsequent predictor models used STARTS = 0 and reproduced the retained solution log likelihood exactly (LL = −21131.809).

Table S5B. Targeted trajectory-robustness checks.

Robustness check	Result	Interpretation / status
Primary trajectory eligibility	N=1129; ≥2 nonmissing w1–w6 CES-D assessments	Retained three-class quadratic FULL GMM

Relaxed trajectory eligibility	N=1146; ≥1 nonmissing w1–w6 CES-D assessment; LL=-21189.554; entropy=.832	Three profiles reproduced closely
Two-class FULL GMM	Inadmissible	PSI/quadratic-factor problems
Two-class diagonal variance specification	Admissible	Targeted boundary robustness
Two-class random-intercept/random-slope with fixed quadratic variance	Admissible	Targeted boundary robustness
Three-class FULL GMM	Admissible	Reproduced established three-profile structure

Table S6. Class-specific growth-factor means for the retained three-class quadratic FULL GMM.

Profile	Parameter	Estimate	SE	p	95% CI
Low-stable	Intercept	10.230	.448	<.001	[9.352, 11.107]
Low-stable	Linear slope	-.323	.235	.170	[-.783, .138]
Low-stable	Quadratic slope	.013	.044	.775	[-.074, .099]
High-decreasing	Intercept	31.173	1.574	<.001	[28.089, 34.258]
High-decreasing	Linear slope	-6.799	1.415	<.001	[-9.571, -4.026]
High-decreasing	Quadratic slope	.822	.260	.002	[.312, 1.331]
Increasing/high-escalating	Intercept	21.749	1.310	<.001	[19.182, 24.315]
Increasing/high-escalating	Linear slope	1.584	.917	.084	[-.213, 3.381]
Increasing/high-escalating	Quadratic slope	.041	.229	.859	[-.408, .489]

Note. Growth-factor variances and covariances were shared across classes.

Table S7. Full R3STEP predictors of depressive symptom profile membership.

Comparison	Predictor	Model 1 OR [95% CI]; p	Model 2 OR [95% CI]; p
High-decreasing vs low-stable	Self-critical perfectionism (z)	3.47 [2.48, 4.87]; <.001	1.92 [1.30, 2.81]; .001
High-decreasing vs low-stable	Personal standards perfectionism (z)	0.77 [0.57, 1.02]; .072	1.11 [0.74, 1.66]; .608
High-decreasing vs low-stable	Age	1.16 [0.83, 1.62]; .386	1.24 [0.83, 1.84]; .288
High-decreasing vs low-stable	Sex: male vs female	0.79 [0.42, 1.51]; .481	1.00 [0.47, 2.13]; .990
High-decreasing vs low-stable	Urban vs rural source	1.20 [0.64, 2.25]; .566	1.67 [0.75, 3.72]; .206
High-decreasing vs low-stable	Highest parental education	1.15 [0.85, 1.55]; .380	1.16 [0.80, 1.69]; .426
High-decreasing vs low-stable	Baseline CES-D	—	1.26 [1.18, 1.33]; <.001
Increasing/high-escalating vs low-stable	Self-critical perfectionism (z)	3.13 [2.32, 4.23]; <.001	1.79 [1.28, 2.49]; .001
Increasing/high-escalating vs low-stable	Personal standards perfectionism (z)	0.90 [0.65, 1.26]; .549	1.44 [0.89, 2.31]; .134
Increasing/high-escalating vs low-stable	Age	1.54 [1.12, 2.12]; .008	1.74 [1.10, 2.74]; .017
Increasing/high-escalating vs low-stable	Sex: male vs female	0.39 [0.21, 0.70]; .002	0.47 [0.22, 1.01]; .052

Comparison	Predictor	Model 1 OR [95% CI]; p	Model 2 OR [95% CI]; p
Increasing/high-escalating vs low-stable	Urban vs rural source	0.67 [0.37, 1.22]; .192	1.14 [0.52, 2.53]; .741
Increasing/high-escalating vs low-stable	Highest parental education	1.62 [1.13, 2.31]; .008	1.72 [1.07, 2.76]; .025
Increasing/high-escalating vs low-stable	Baseline CES-D	—	1.25 [1.18, 1.32]; <.001

Note. Reference profile = low-stable. Model 1 N = 1033; Model 2 N = 1022. Both models reproduced the retained trajectory-model log likelihood exactly with STARTS = 0.

Table S8. Direct contrasts of self-critical versus personal-standards coefficients.

Model	Class contrast	B_SC	B_PS	ΔB (SC−PS)	SE(ΔB)	Wald χ^2	p	95% CI
Model 1	High-decreasing vs low-stable	1.245	-.267	1.512	.240	39.740	<.001	[1.042, 1.982]
Model 1	Increasing/high-escalating vs low-stable	1.140	-.100	1.241	.250	24.711	<.001	[.752, 1.730]
Model 2	High-decreasing vs low-stable	.650	.105	.545	.301	3.280	.070	[-.045, 1.135]
Model 2	Increasing/high-escalating vs low-stable	.579	.362	.217	.303	.513	.474	[-.377, .810]

Note. Exact parameter covariances were used. Manual three-step reproduction of automatic R3STEP focal coefficients passed; maximum absolute B difference=.001 and maximum absolute SE difference=.000.

Table S9. Targeted sensitivity analysis excluding parental education.

Model	Class contrast	Predictor	Main B	Main OR	Sensitivity B	Sensitivity OR	ΔB	Direction	p: main → sensitivity
M1	High-decreasing vs low-stable	SC	1.245	3.473	1.305	3.689	+.060	Stable	<.001 → <.001
M1	Increasing/high-escalating vs low-stable	SC	1.141	3.130	1.139	3.122	-.002	Stable	<.001 → <.001
M2	High-decreasing vs low-stable	SC	.650	1.916	.722	2.059	+.072	Stable	.001 → <.001
M2	Increasing/high-escalating vs low-stable	SC	.579	1.784	.599	1.820	+.020	Stable	.001 → <.001
M1	High-decreasing vs low-stable	PS	-.267	.766	-.298	.742	-.031	Direction stable	.072 → .049
M1	Increasing/high-escalating vs low-stable	PS	-.100	.905	-.113	.893	-.013	Stable	.549 → .497

Model	Class contrast	Predictor	Main B	Main OR	Sensitivity B	Sensitivity OR	ΔB	Direction	p: main → sensitivity
M2	High-decreasing vs low-stable	PS	.105	1.111	.043	1.044	-.062	Stable	.608 → .821
M2	Increasing/high-escalating vs low-stable	PS	.362	1.436	.284	1.329	-.078	Stable	.134 → .196

Note. Sensitivity Model 1 N=1100; Model 2 N=1088. Maximum absolute change among the four self-critical coefficients=.072; maximum absolute change across all eight focal SC/PS coefficients=.078. Both sensitivity models reproduced the retained trajectory-model log likelihood (LL = -21131.809).

Table S10. Sensitivity analysis excluding DPSCA14 from the self-critical perfectionism score.

Model	Class contrast	Main 7-item B	6-item B	ΔB	Main OR	6-item OR	p: main → 6-item
Model 1	High-decreasing vs low-stable	1.245	1.310	+.065	3.473	3.705	<.001 → <.001
Model 1	Increasing/high-escalating vs low-stable	1.141	1.139	-.002	3.130	3.123	<.001 → <.001
Model 2	High-decreasing vs low-stable	.650	.718	+.068	1.916	2.050	.001 → <.001
Model 2	Increasing/high-escalating vs low-stable	.579	.582	+.003	1.784	1.790	.001 → .001

Note. The six-item sensitivity score excluded DPSCA14 and retained DPSCA4, 6, 7, 8, 10, and 12; scores were computed when at least five of six items were available as mean(valid) × 6 and standardized once in the baseline scoring-eligible sample. Model 1 N = 1033 and Model 2 N = 1022, identical to the primary analyses. All four self-critical coefficients retained their direction and significance status. The maximum absolute ΔB was .068 and the maximum relative ΔB was 10.462%. The six-item score showed raw α = .609, standardized α = .627, and ω = .638. Both sensitivity models reproduced the retained trajectory-model log likelihood exactly (LL = -21131.809; STARTS = 0). The original seven-item self-critical score remained the prespecified primary score.

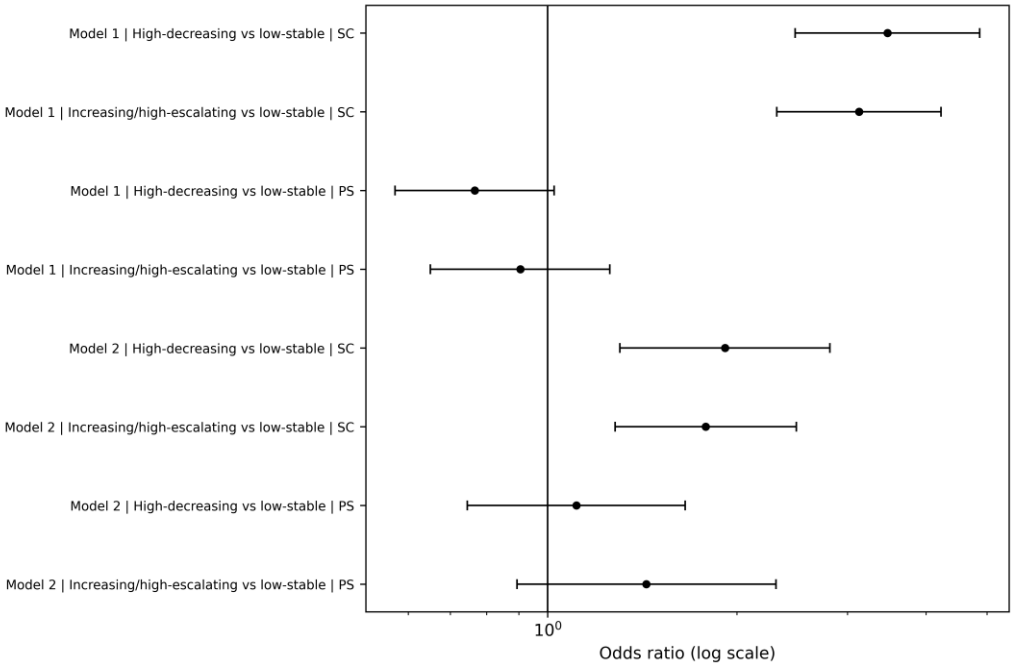

Figure S1. Perfectionism dimensions predicting depressive symptom profile membership in Model 1 and Model 2.

Note. Points show odds ratios and horizontal bars show 95% confidence intervals from the main two-predictor R3STEP models. SC = self-critical perfectionism; PS = personal standards perfectionism. The reference profile was low-stable.
